# Oyster cooking practices in the United States-based restaurants—A survey

**DOI:** 10.1371/journal.pone.0327330

**Published:** 2025-07-16

**Authors:** Razieh Sadat Mirmahdi, Razieh Farzad, Andrew J. MacIntosh, Arie H. Havelaar, Amarat H. Simonne, Naim Montazeri

**Affiliations:** 1 Food Science and Human Nutrition Department, University of Florida, Gainesville, Florida, United States of America; 2 Animal Sciences Department and Emerging Pathogens Institute, University of Florida, Gainesville, Florida, United States of America; 3 Global Food Systems Institute, University of Florida, Gainesville, Florida, United States of America; 4 Family, Youth and Community Sciences Department, University of Florida, Gainesville, Florida, United States of America; Lusofona University of Humanities and Technologies: Universidade Lusofona de Humanidades e Tecnologias, PORTUGAL

## Abstract

Despite longstanding oyster cooking recommendations, outbreaks associated with cooked oysters still occur. A survey of U.S.-based restaurants was conducted to investigate common cooking practices, including steaming, baking, and roasting. Target restaurants were identified using Standard Industrial Classification (SIC) codes and surveyed through live phone interviews and online. The questionnaire included open- and closed-ended questions for restaurant staff, including chefs and managers, with topics covering customer and serving quantities, source of purchase, common cooking methods, cooking time and temperature combinations, and the use of thermometers. A total of 105 complete responses were collected from California, Florida, Louisiana, Massachusetts, Oregon, Virginia, and Washington. On a weekly basis, the majority of restaurants served 1–1,000 customers with 1–500 dozen oysters. The most frequently used cooking methods were frying (46%), followed by baking (36%), steaming (30%), and then roasting (23%). On average, baking was performed at a temperature of 185 ± 64°C for 9 ± 4 minutes, roasting at 207 ± 54°C for 8 ± 6 minutes, and steaming for 5 ± 3 minutes, with no correlation being found between cooking time and temperature for either technique. Additionally, 57% of the surveyed restaurants did not use thermometers when cooking oysters. This study highlights the variations in oyster cooking practices in U.S. restaurants, emphasizing the need to assess the effectiveness of different cooking techniques through quantitative microbial risk assessment of the most common pathogens in oysters. This will help improve food safety guidelines and minimize health risks associated with the consumption of partially cooked oysters.

## Introduction

Oysters are prone to contamination with pathogens when cultivated in polluted waters. It is due to their filter-feeding behavior, which leads to the accumulation of microorganisms in various tissues, especially digestive diverticula [1–3]. Oysters sourced from “approved” areas carry the lowest risk of pathogen contamination [4]. Given the potential for oyster contamination, the United States (U.S.) Centers for Disease Control and Prevention (CDC) cautions against eating raw or undercooked oysters and emphasizes the importance of avoiding cross-contamination by keeping raw oysters, their drippings, or juices away from other foods and surfaces [5]. The risk of infection is highest for vulnerable and susceptible subpopulations, including children, the elderly, and immunocompromised individuals [6,7]. According to the U.S. CDC BEAM (Bacteria, Enterics, Ameba, and Mycotics) Dashboard, a total of 296 outbreaks related to oyster consumption have been reported in the U.S. between 1971 and 2023. Among U.S. states, Washington recorded the highest number of outbreaks with 85, followed by California with 51, Florida with 26, and Oregon with 23 outbreaks. The pathogens assosiated with the outbreaks were mostly norovirus, *Salmonella*, sapovirus, *Vibrio*, and *Campylobacter*. Ten outbreaks, 114 illnesses, and one hospitalization were confirmed to be linked to the consumption of cooked oysters prepared using frying, baking, and steaming methods in the U.S. [8].

Guidelines provided by the U.S. CDC specify temperature and time recommendations for cooking in-shell and shucked oysters, specifically targeting *Vibrio* spp. [5]. Accordingly, boiling in-shell oysters is recommended until the shells open, and continue cooking for an additional 3–5 minutes. Alternatively, steam cooking should be conducted for 4–9 minutes. For shucked oysters, the U.S. CDC recommends cooking by boiling for at least 3 minutes, frying in oil at 190°C for at least 3 minutes, broiling from a distance of 7.5 cm from the heat source for 3 minutes, or baking at 230°C for 10 minutes. Globally, the guidelines for cooking oysters vary considerably. For instance, the Canadian British Columbia Centre for Disease Control (BCCDC) does not recommend specific times for preparation but instead recommends preparing the shellfish to an internal temperature of 90ºC for 90 seconds [9]. While likely effective, the BCCDC guidelines are less useful for small-scale producers who may have difficulty tracking the internal temperature of their products.

Despite cooking recommendations for oysters, outbreaks involving cooked oysters still occur. While it is not clear whether the majority of these incidents occurred at homes or restaurants, they indicate deviations from following the recommended time and temperature combinations for a particular cooking technique. Limited data is available on the common oyster cooking techniques. A survey on oyster consumers’ preferences in South Carolina identified steaming as the most popular cooking method, followed by grilling, consuming raw oysters, and incorporating them into recipes with other ingredients, such as soups and stews. Additionally, they showed that 75% of the consumers were willing to eat oysters at restaurants, 44% at home, and 41% at oyster roasts — a Southern tradition of cooking oysters over a fire [10]. 

There is currently no information on the cooking practices for oysters in U.S. restaurants, such as time, temperature, or whether they are cooked in the shell or half-shell. To identify current cooking conditions, we surveyed techniques used in restaurants rather than those used in home cooking. Additionally, based on the U.S. CDC report on outbreaks from partially cooked oysters, we selected steaming and baking as the primary methods for this study. The roasting method was also included due to the limited data on this technique in the literature, highlighting the need for further investigation. The findings provide a baseline for assessing the effectiveness of these techniques in inactivating foodborne pathogens in oysters and contribute to refining quantitative risk assessment models for enhancing the microbial safety of oysters.

## Materials and methods

### Research population

The targeted population for this survey consisted of U.S.-based restaurants that serve oysters. Restaurants were identified using the established Standard Industrial Classification (SIC) Codes to select categories of restaurants that are more likely to feature oysters on their menu [11]. Only restaurants with the following SIC codes were selected: 58120102 (Cajun restaurant), 58120700 (Seafood restaurant), 58120701 (Oyster bar), and 58120702 (Seafood shack). Records for restaurants with the correct SIC codes were pulled from California, Florida, Louisiana, Massachusetts, Oregon, Virginia, and Washington. No additional criteria were used to include or exclude restaurants from the list. Consequently, two sample files were provided: one with email addresses containing 799 records and one with physical addresses/phone numbers containing 2,973 records. Regarding the phone survey, there were 3,506 total phone records (which include online samples ported into the phone survey).

### Questionnaire design and distribution

Given the variety of approaches for cooking oysters, this study was mainly focused on steaming, baking, and roasting techniques used by U.S.-based restaurants, as defined in Table 1. The interviews were directed at restaurant staff, including chefs, managers, and other employees familiar with the restaurant’s cooking practices, with the screening criterion requiring the participants to be 18 years old or older. The questionnaire included open-ended and close-ended questions (Table 2), with response categories based on a preliminary review of the literature. Following an initial pre-test, the interviews were conducted by asking about confidence in cooking oysters safely, consumer and serving quantities, sources for purchasing oysters, common cooking practices, cooking time and temperature for steaming, roasting, or baking, how they determine when oysters are ready to serve, how many steamed, baked, and roasted oysters they serve weekly, whether thermometers are used, and various demographic questions.

**Table 1:** Definition of the cooking methods provided to participants.

Cooking techniques^a^	Definition
**Roasting**	Place oysters in a single layer over an open flame or on a grill.
**Baking**	Place oysters in a single layer in an enclosed environment such as an oven.
**Steaming**	Place the half-shell or whole oysters in a steamer with boiling water, covering them, then steaming until done.

a Cooking techniques are defined as the preparation of oysters just before consumption.

**Table 2:** Survey questionnaire on oyster preparation practices in U.S.-based restaurants^*^.

Serving and preparation questions	Cooking techniques questions	Demographic questions
A.1. What number would you use to rate this statement: Preparing oysters (by steaming, baking, or roasting) makes them safe to consume (using any number from 0 to10, where 0 is not at all confident and 10 is very confident.) Enter amount:	B.1. What techniques do you use to prepare oysters? You may choose all that apply. 1 Steaming 2 Baking 3 Roasting 4 Other (Please specify)	C.1. How would you describe your gender? 1 Male 2 Female 3 Non-binary/third gender 4 I prefer not to self-describe
A.2. How many customers eat at your restaurant in an average week? Enter amount:	B.2. Approximately how many servings of oysters do you STEAM per week? Enter amount:	What is your age? Enter amount:
A.3. On average, how many cooked (dozens of) oysters do you usually serve to consumers in a week? Enter amount:	B.3. When STEAMING oysters, do you usually use oysters in the half-shell, whole, or with no shell? 1 Half shell 2 Whole shell 3 No shell	What are the FIRST THREE DIGITS of your establishment’s zip code? Zip code:
A.4. Where do you purchase most of the oysters prepared in your establishment? 1 Local grocery store 2 Seafood market 3 Directly from a farm or harvester 4 Online 5 Other (Please specify):	B.4. From the time the pot starts boiling, for about how many minutes do you STEAM oysters? Enter amount:	
	B.5. How do you assess whether STEAMED oysters are ready to serve? (When do you consider them “done”?) Consider aspects like texture, color, juiciness, opacity, smell, and shell openness. Response:	
	B.6. Approximately how many servings of oysters do you BAKE per week? Enter amount:	
	B.7. When BAKING oysters, do you usually use oysters in half-shell, whole, or with no shell? 1 Half shell 2 Whole shell 3 No shell	
	B.8. At what temperature do you typically BAKE oysters? Please give your answer in Fahrenheit (°F). Enter amount:	
	B.9. About how long does it typically take you to BAKE oysters? Please give your time in minutes. Enter amount:	
	B.10. How do you assess whether BAKED oysters are ready to serve? (How do you consider them “done”). Consider aspects like texture, color, juiciness, opacity, smell, and shell openness. Response:	
	B.11. Approximately how many servings of oysters do you ROAST per week? Enter amount:	
	B.12. When ROASTING oysters, do you use oysters in half-shell, whole, or with no shell? 1 Half shell 2 Whole shell 3 No shell	
	B.13. At what temperature do you typically ROAST oysters? Please give your answer in Fahrenheit (°F). Enter amount:	
	B.14. About how long does it typically take you to ROAST oysters? Please give your time in minutes. Enter amount:	
	B.15. How do you assess whether ROASTED oysters are ready to serve? Consider aspects like color, juiciness, opacity, smell, and shell openness. Response:	
	B.16. Please identify whether you use a thermometer to measure the internal temperature of the oysters when steaming, baking, or roasting oysters. 1 Steaming 2 Baking 3 Roasting 4 THERMOMETER NOT USED	
	B.17. Are there any other details about preparing oysters that we haven’t asked you about that might be important for us to know? This can include thermometer use, cookware, spices (like butter, garlic, cheese), or other details Response:	

* In all questions, ‘-8’ represents ‘don’t know’ and ‘-9’ represents ‘refused’.

The survey was conducted by the University of Florida Survey Research Center (UFSRC) using two methods: online (self-administered) through the Qualtrics platform and phone (administered by trained UFSRC telephone interviewers). Prior to launching the surveys, ethical permissions were obtained from the University of Florida Institutional Review Board (UF IRB), which reviewed and approved the study protocol (Approval Number: 202300297) in accordance with ethical guidelines for research involving human subjects. The participants were presented with an informed consent statement immediately prior to the beginning of the survey.

For the phone survey, the operator explained the aim of the study and informed respondents that all responses all optional. If they chose to participate, they were informed that they had the right to refuse to answer specific questions or to end the interview at any time. Additionally, they were assured that their responses would be kept confidential and that their names would not be linked to any of the information provided. For the online survey, respondents were provided with a consent form. It informed participants that completing and submitting the survey implies that they had read the information and consented to participate in the study. As the data were analyzed anonymously, UF IRB approved the waiver of documentation of informed consent for subjects participating in this survey.

The online survey was launched on April 10, 2024, and data collection ended on May 30, 2024. The initial invitation was sent on April 10^th^, with three reminders sent approximately one week after the other. One final reminder was sent on May 28. A total of 799 online invitations were sent, with 223 returned as undelivered and 88 reminder messages failed to reach the participants. From the emails that were successfully sent, the UFSRC established 23 online surveys, of which 11 were fully completed.

The phone survey was conducted from April 29 to May 30, 2024. The UFSRC specified certain times of day to call to limit phone call attempts to times that are not typically “peak hours” for restaurants. The U.S.-based restaurants were called Sunday through Saturday, 9–11 AM, 1:30–5:30 PM, and 8–9 PM according to the establishment’s local time zones. Up to three phone call attempts were made for each establishment; however, the number of call attempts could go higher if a respondent requested a callback on what would be the final attempt. Based on respondent requests, records were dialed up to 11 times. Toward the end of the survey fielding period, 533 additional phone records were added to the sample. Of the 3,506 total phone records (which includes an online sample ported into the phone survey), 464 were disconnected or non-functional, and 334 establishments indicated that they did not serve or no longer offered oysters. Ultimately, UFSRC interviewers completed 94 phone interviews. The information collected from the surveys was de-identified, and the Qualtrics “anonymization” feature was used so responses could not be connected to email addresses. Anonymous survey data (with demographic and location information removed), along with the questionnaire codebook, are publicly available on Zenodo (https://doi.org/10.5281/zenodo.15347152).

### Data analysis

Data was refined by removing any identifiers, blank fields, and incomplete responses. The collected data are reported as mean ± standard deviation. The response frequencies are presented as proportions and visualized accordingly. The relationship between time and temperature for each cooking technique was analyzed using the Pearson correlation coefficient and linear regression. Statistical analyses and visualizations were conducted using RStudio version 4.4.2 [12], and open-source packages, including readxl [13], ggplot2 [14], dplyr [15], RColorBrewer [16], and webr [17]. Approximate locations of the surveyed restaurants, based on their 3-digit ZIP codes, were obtained from the U.S. Census Bureau database [18]. The U.S. state boundary map was derived from the U.S. Census Bureau’s Cartographic Boundary database [19] and adapted using ArcGIS Online (https://ufl.maps.arcgis.com), hosted by the University of Florida GeoPlan Center (Gainesville, FL). Locations were plotted using the ggplot2 package in R [14], and final figure overlays and enhancements were performed using the online draw.io platform (https://www.drawio.com).

## Results and discussion

### Survey administration and participant demographics

In total, 105 complete survey responses (11 online and 94 phone surveys) were collected. The majority (65%, 68 out of 105) of respondents were male, and 49% (51 out of 105) were between 30 and 50 years (Table 3).

**Table 3:** Demographic characteristics of the respondents.

Demographic variable	N (% of the respondents) (total n = 105)
**Gender**	
**Male**	68 (65%)
**Female**	32 (30%)
**Did not disclose**	5 (5%)
**Age**	
**< 30 years**	14 (13%)
**30- 50 years**	51 (49%)
**50- 80 years**	35 (33%)
**Did not disclose**	5 (5%)

**Fig 1:**
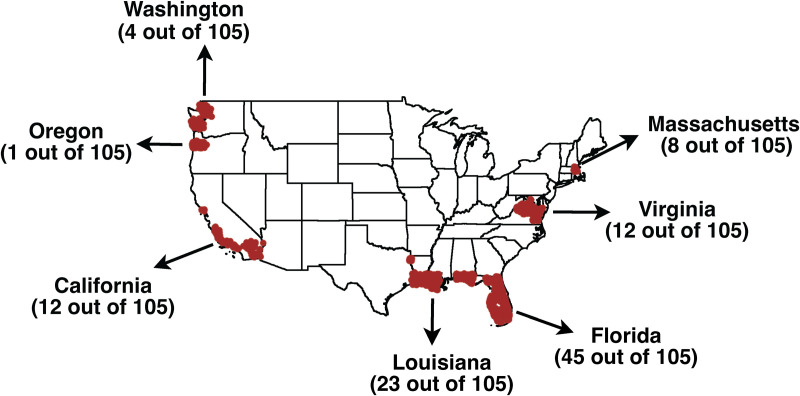
Geographic distribution and response rates of surveyed restaurants. The marked areas indicate the approximate locations of the restaurants where the survey was distributed in seven U.S. states: California, Florida, Louisiana, Massachusetts, Oregon, Virginia, and Washington. The percentages and numbers show the responses received from each state out of a total of 105 complete responses. The U.S. outline map was adapted from the publicly accessible U.S. Census Bureau database [19].

Responses were received from restaurants across seven U.S. states, California, Florida, Louisiana, Massachusetts, Oregon, Virginia, and Washington. The geographic distribution of the survey locations is illustrated in Fig 1. The survey results indicate that a significant portion of the responses originated from Florida, accounting for 43% (45 out of 105) of the total responses collected, followed by Louisiana with 22% of responses (23 out of 105, Fig 1). Based on annual gross sales, Louisiana and Florida, along with Connecticut, Massachusetts, and Maine, were the top five states in 2023 with regard to the production of Eastern oysters [20].

Our survey findings closely align with a report on the regional oyster consumption habits in 2000–2001 [21]. The report indicated that the highest percentage of consumers was in the East South-Central region (Kentucky, Mississippi, Tennessee, and Alabama) at 56% (n = 133), followed by the West South-Central region (Texas, Arkansas, Louisiana, and Oklahoma) at 54% (n = 140), the Southeast Atlantic region (Florida, Georgia, North Carolina, South Carolina, West Virginia, Virginia, Maryland, Delaware, and Washington, D.C.) at 53% (n = 153), and the Pacific region (Alaska, Hawaii, California, Oregon, and Washington), where 47% (n = 143) of respondents consumed oysters [21].

### Serving and preparation of oysters

The first step in ensuring the safety of oysters is purchasing them from approved sources [22]. The primary source of oysters purchased by the surveyed restaurants (Table 2, Question A.4) was directly from a farm or harvester (58%, 61 out of 105), followed by seafood markets (11%, 12 out of 105). Other sources (29%, 31 out of 105) include oyster processing companies, fish sellers and buyers, seafood supply companies, food service companies, and vendors (S1 Table). The next crucial step in ensuring oyster safety is proper handling and storage. Oysters should be kept in clean, cool containers to prevent contamination during handling. If the oysters are to be consumed within two days, they should be refrigerated immediately. Otherwise, they must be tightly wrapped and stored in the freezer to prevent spoilage. Inadequate storage can increase the risk of contamination by common bacteria such as *Arcobacter*, *Spirochaeta*, *Pseudoalteromonas*, *Marinomonas*, *Fusobacterium*, *Psychrobacter*, *Psychromonas*, and *Oceanisphaera* [4,23]. Regarding restaurants’ confidence in the safety of oysters after cooking (Table 2, Question A.1), respondents were asked to rate their confidence on a scale from 1 to 10, with an average response of 8.4 ± 2.3. Results showed that 40% (42 out of 105) of the respondents rated their confidence as 10, indicating they were very confident in the safety of cooked oysters. An additional 44% (46 out of 105) rated their confidence between 6 and 9, while 13% (14 out of 105) rated their confidence at 5 or lower.

While data on restaurant confidence in oyster safety is limited, more research has been focused on consumer perceptions of oyster safety. Understanding these perceptions is crucial, as it could influence purchasing decisions and overall oyster consumption patterns. Personal preferences and regional residency influence the perception that oysters from their areas are safer [21]. They also indicated that among the seven regions producing oysters, including the Pacific Northwest, Gulf of Mexico, Chesapeake Bay, New England, Southeast Atlantic, and Mid-Atlantic, the majority of oyster consumers (65%) had no opinion on which region has the safest oysters (including both oyster consumers and non-consumers). Most of the oyster consumers considered Pacific Northwest oysters to be the safest, followed by New England oysters. However, these perceptions were influenced by the region of residence and personal preferences. For example, 42% of Pacific Northwest residents believed that oysters from their region were the safest source, while the remaining respondents had no opinion on the matter [21].

**Fig 2:**
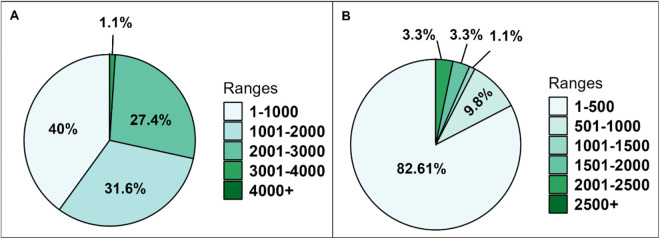
Weekly number of customers dining at surveyed restaurants (A), Weekly dozens of cooked oysters served at surveyed restaurants (B).

The majority of restaurants (40%, 42 out of 105) reported having 1–1,000 weekly customers, 31.6% (33 out of 105) with 1,001–2,000 customers, 27.37% (29 out of 105) with 2,001–3,000 customers, and 1.1% (1 out of 105) with 3,001–4,000 customers weekly (Fig 2A). The total number of oysters (both raw and cooked) served weekly in the surveyed restaurants was distributed as follows: 83% (76 out of 105) served 1–500 dozen, 10% (9 out of 105) served 501–1,000 dozen, 1% (1 out of 105) served 1,001–1,500 dozen, 3% (3 out of 105) served 1,501–2,000 dozen, and 3% (3 out of 105) served 2,001–2,500 dozen of oysters weekly (Fig 2B). For establishing the risk assessment models, the frequency of oyster consumption by each individual also matters. In 1994, a phone survey was conducted across North and South Florida, asking 471 individuals about their oyster consumption frequency over the past year. The results showed that 152 individuals (32%) did not consume oysters. Among the remaining respondents, 46 (10%) consumed oysters once during the year, 161 (34.2%) once or twice in the past six months, 48 (10%) once per month, 36 (8%) twice a month, 8 (3%) three times per month, 15 (3%) four times a month, and 5 (1%) more than once a week [24].

### Oyster cooking techniques and serving numbers

**Fig 3:**
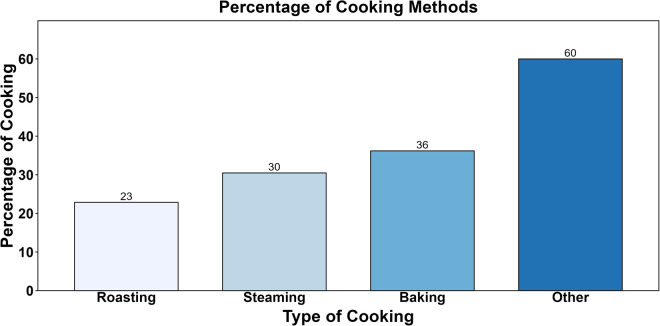
Percentage of oyster cooking practices used by surveyed restaurants.

Of 105 restaurants, 38 reported baking, 32 reported steaming, and 24 preferred roasting as their oyster cooking techniques (Table 2, Question B.1). Fig 3 illustrates the percentage of each cooking practice used in the surveyed restaurants. Additionally, 63 out of 105 respondents mentioned using other cooking methods, including frying (48 out of 105, 46%) and boiling (1 out of 105, 1%, Table 4). The respondents were allowed to select multiple cooking techniques, resulting in a cumulative percentage exceeding 100%.

**Table 4:** The other cooking techniques used by the surveyed restaurants.

Other cooking techniques	Number of restaurants (N = 105)	Percentage
**Frying**	48	46%
**Boiling**	1	1%
**Grilling/Chargrilled/charred/ broil/ Broil-mesquite/ Charbroiled/blackened**	12	11%
**Raw/non**	13	12%

The bar graph shows the distribution of oyster cooking methods used by the surveyed restaurants, highlighting the popularity of each cooking technique. Others include frying, grilling, and boiling. Respondents could select multiple techniques, leading to a total percentage exceeding 100%.

A similar survey examining consumer preferences for oyster consumption in South Carolina reported that a majority of individuals preferred cooked oysters over raw ones, with steamed oysters being the preferred choice (71%), followed by grilled oysters (48%), raw oysters (42%), and cooked oysters in the forms of soups and stews (33%). Among raw oyster consumers, 73% expressed little to no concern about the safety of consuming raw oysters [10]. In another survey conducted in Delaware, oyster consumers were asked about their preferences for purchasing prepared oysters. Fried oysters were the most favored option by 58% of the participants, followed by raw oysters on the half-shell at 28% and take-home whole oysters on ice at 14% [25].

**Fig 4:**
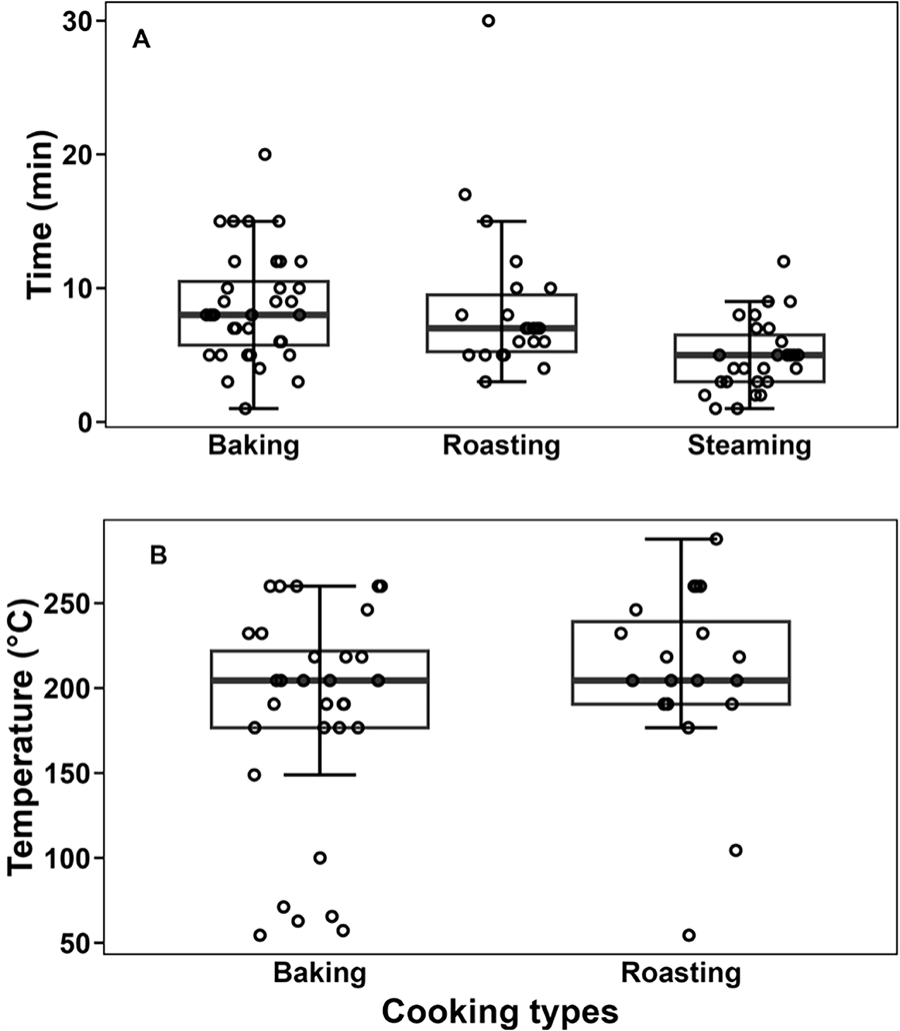
Time and temperature used for different oyster cooking techniques. Box plots illustrate the cooking times (A) and temperatures (B) restaurants reported to use for various oyster cooking techniques. The temperature for steaming was assumed to be 100ºC (not shown in plot B). The upper line in each box represents the third quartile, the middle line indicates the median, and the lower line corresponds to the first quartile.

Of the 32 restaurants that served steamed oysters, 26 of them reported serving sizes, with 58% (15 out of 26) reported 1–50 servings, 8% (2 out of 26) reported 51–100 servings, 4% (1 out of 26) reported 151–200 servings, and 31% (8 out of 26) reported more than 200 servings per week. Restaurants used both half-shell and whole-shell oysters for steaming, with 52% (16 out of 31) opting for half-shell and 48% (15 out of 31) choosing whole-shell oysters. For steaming, the cooking temperature was assumed to be 100°C —the temperature of boiling water at a standard atmospheric pressure. As shown in Fig 4, the mean steaming time was 4.88 ± 2.68 minutes (n = 27), which aligns with the CDC’s recommendation of steaming oysters for 4–9 minutes.

The oyster’s texture (47%, 14 out of 30), shell openness (40%, 12 out of 30), color (30%, 9 out of 30), appearance (20%, 6 out of 30), juiciness (17%, 5 out of 30), opacity (17%, 5 out of 30), and smell (17%, 5 out of 30) were the most used factors in determining when steamed oysters were considered as ready to serve. This was an open-ended question, and the details of the responses are included in the supplementary material (S2 Table).

For baking, 31 respondents provided information on their weekly servings of baked oysters. Among them, 45% (14 out of 31) reported 1–50 servings, followed by 35% (11 out of 31) with 51–100 servings, 3% (1 out of 31) with 101–150 servings, 3% (1 out of 31) with 151–200 servings, and 13% (4 out of 31) with more than 200 servings weekly. For baking, restaurants primarily used half-shell oysters (84%, 32 out of 38), followed by whole oysters (11%, 4 out of 38), and no-shell oysters (5%, 2 out of 38). For baking, the mean cooking time was 8.6 ± 4.1 minutes, and the mean temperature was 185.0 ± 63.9°C (n = 32, Fig 4). Six respondents reported using baking temperatures equal to or less than 100°C, which were considered leverage points as they considerably influenced the central tendency and dispersion of cooking temperatures. The following assumptions were made regarding this observation. Firstly, we asked respondents to report the temperature in ºF, but they might have misreported it as °C, as their reported values closely align with other data in °C. Another assumption is that they reported the internal temperature instead of the external one. A limitation of this study is that the questionnaire did not specify whether the reported temperatures refer to internal or external measurements for each cooking technique. The recalculated baking temperature, after excluding the leverage points, was 211.9 ± 31.5°C, and the new box plot of the temperature is shown in the supplementary material (S1 Fig). The recalculated mean baking temperature is closer to the U.S. CDC recommendation, which specifies that shucked oysters should be baked at 232ºC for 10 minutes, ensuring they reach an internal temperature of 62.8ºC [5].

The oyster’s texture (44%, 16 out of 36), color (33%, 12 out of 36), shell openness (19%, 7 out of 36), juiciness (19%, 7 out of 36), appearance (17%, 6 out of 36), and internal temperature (17%, 6 out of 36) are the most used factors in determining when baked oysters are ready to serve. This was an open-ended question, and the details of the responses are included in the supplementary material (S2 Table).

Overall, 19 respondents provided information on their weekly servings of roasted oysters. Among them, 68% (13 out of 19) reported 1–50 servings, followed by 16% (3 out of 19) with 51–100 servings, and 13% (3 out of 19) with more than 200 servings weekly. For roasting, restaurants primarily used half-shell oysters (86%, 19 out of 22), followed by whole oysters (9%, 2 out of 22), and no-shell oysters (5%, 1 out of 22). The mean time and temperature for roasting oysters were 8.26 ± 6.01 minutes and 207.4 ± 54.6°C (n = 19), respectively (Fig 4A, B). One of the respondents reported using less than 100°C for baking oysters, which was considered a leverage point, as explained earlier. After excluding the leverage point, the recalculated roasting temperature was 215.9 ± 41.2°C, and the new box plot of the temperature is shown in the supplementary material (S1 Fig). The U.S. CDC has not provided any time and temperature recommendations for roasting oysters, highlighting the need for further research.

The oyster’s texture (36.4%, 8 out of 22), color (36.4%, 8 out of 22), juiciness (22.7%, 5 out of 22), appearance (18.2%, 4 out of 22), and internal temperature (13.6%, 3 out of 22) are the most used factors in determining when roasted oysters are ready to serve. As a few restaurants use a thermometer as a readiness factor for oysters, we do not have information on whether their reported cooking temperature refers to internal or external measurements. This was an open-ended question, and the details of the responses are included in the supplementary material (S2 Table).

**Fig 5:**
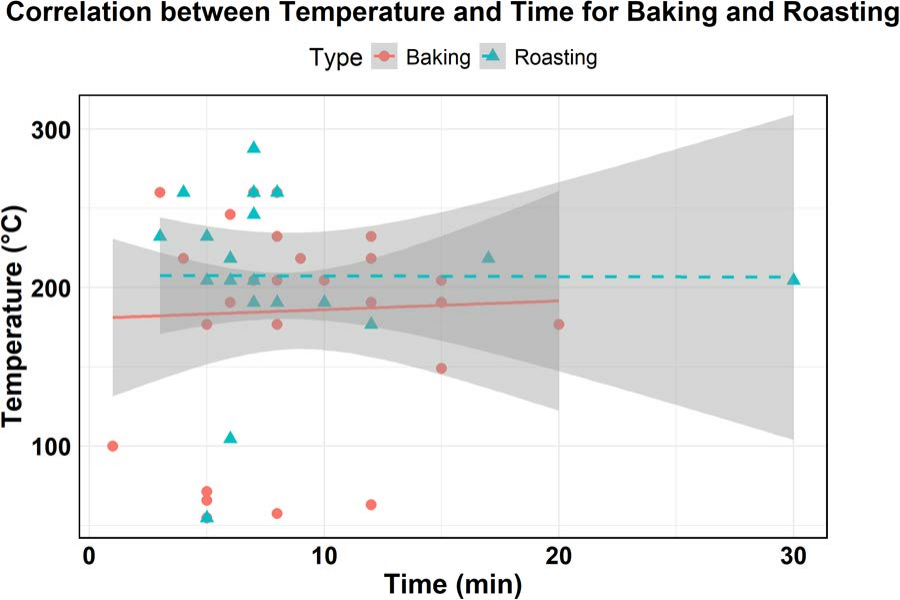
The relationships between time and temperature of baking and roasting techniques. The lines indicate the linear fit, and the 95% confidence intervals are presented as gray areas around each fitted line.

To assess the relationship between time and temperature across different cooking methods, correlation analyses were performed separately for both roasting and baking techniques. No noticeable correlation was observed in the collective data between the time and temperature used for roasting (*r* = −0.004) or baking (*r* = 0.034, Fig 5). Once the correlations were reestablished following the removal of the leverage points, the time and temperature showed moderate correlations of *r* = −0.129 for roasting and *r* = −0.562 for baking techniques (S2 Fig). This suggests that the presence of leverage points initially masked the underlying trends, particularly in baking, where a stronger inverse relationship between time and temperature was observed. The lack of strong correlation between cooking time and temperature suggests the need to establish standardized procedures for cooking oysters. This variability might show that there is currently no consistent protocol being followed in restaurants. Developing and implementing standardized cooking guidelines would benefit by reducing the risk of foodborne illness and strengthening consumer confidence in the safety and quality of the cooked oysters.

Regarding the use of thermometers to measure the internal temperature of oysters (Table 2, Question B.16), 34.4% (11 out of 32) of restaurants reported using thermometers for steaming, 50.0% (19 out of 38) used them during baking, and 50.0% (12 out of 24) utilized thermometers for roasting. Notably, 57% (60 out of 105) did not use thermometers for any cooking method, and 16% (16 out of 105) did not specify whether they used thermometers. Regardless, without a thermometer, estimating the internal temperature and ensuring it reaches the appropriate level for pathogen inactivation can be challenging. The shell of the oysters consists of multiple layers of calcium carbonate within a conchiolin matrix and numerous pores. The complexity of oyster shells leads to variations in thermal conductivity and density among individual oysters [26–28]. Furthermore, since steaming typically involves in-shell whole oysters, measuring internal temperatures may not be practical. Reliance on shell opening does not guarantee that oysters have reached safe cooking temperatures, making it essential to establish adequate cooking temperatures and times for steaming. Despite the variability in achieving the internal temperature among oysters, a study using a thermometer to measure the internal temperature of the oysters estimated the internal temperatures of deep-fried, baked, grilled, and pan-fried oysters to be 76.5°C, 59.4°C, 79.3°C, and 88.8°C, respectively. These temperatures were reached after pan-fried and grilled oysters were cooked for at least 5 minutes, while deep-fried and baked oysters had cooking times ranging from 2 to 15 minutes [29]. Additionally, the size of the oysters, along with the chemical composition, size, and shape of the cooking container, can impact heat transfer rates. Beyond the challenge of achieving an adequate internal temperature in oysters, other factors influencing microbial survival after heat treatment include the distribution of pathogens within the oysters, relative humidity, and the forms in which microbes are present [30].

Targeted education and training are recommended for chefs and kitchen staff involved in oyster preparation in restaurants. Training should emphasize proper handling and storage, prevention of cross-contamination by separating raw oysters and their juices from ready-to-eat foods, and the correct use of food thermometers to ensure reaching a desired time-temperature combination for each cooking technique. The standardized cooking methods are critical not only to reduce the risk of pathogen infection but also to limit the formation of potentially harmful thermal byproducts and preserve the nutrient digestibility and bioaccessibility of oysters. The findings from this study contribute to the development of a more accurate risk assessment framework by providing baseline information for exposure assessment and risk characterization concepts of the risk assessment framework, aimed at reducing the risks associated with foodborne pathogens in undercooked oysters and thereby safeguarding public health.

## Conclusions

This study showed that the primary source of oyster purchases for U.S.-based restaurants is directly from a farm or harvester (58%). Among cooking techniques, frying (46%) was the most used in the restaurants, followed by baking (36%), steaming (30%), and roasting (23%). For steaming, oysters were prepared in both half-shell (48%) and whole-shell (52%) forms, with a mean cooking time of 5 ± 3 minutes. The most important factors for assessing oyster readiness were texture (47%) and shell openness (40%). For baking, half-shell oysters (84%) were primarily used, with a mean cooking temperature of 185 ± 64°C and a mean time of 9 ± 4 minutes. For roasting, restaurants primarily used half-shell oysters (86%), with a mean cooking time of 8 ± 6 minutes and a mean temperature of 207 ± 55°C. The primary factors for assessing readiness in baked and roasted oysters were texture and color changes. There was no correlation between time and temperature across the cooking techniques. Additionally, most restaurants (57%) did not use thermometers for any cooking technique. The findings from this study contribute to the development of an accurate risk assessment framework by providing information for the risk characterization step of the QMRA, aimed at helping to reduce the risks associated with foodborne pathogens in undercooked oysters and thereby safeguarding public health. Further research is needed to determine whether current cooking techniques are sufficient to ensure oyster safety, particularly in mitigating risks for individuals with weakened immune systems.

## Supporting information

S1 Fig Temperature used for different oyster cooking techniques after removing the leverage points (cooking temperatures equal or less than 100°C). Box plots illustrate the cooking temperatures restaurants reported to use for various oyster cooking techniques. The temperature for steaming was assumed to be 100ºC (not shown). The upper line in each box represents the third quartile, the middle line indicates the median, and the lower line corresponds to the first quartile.
(TIFF)

S2 Fig The relationships between time and temperature of baking and roasting techniques are shown by the Pearson correlation coefficients (*r*). Data represents the recalculation of Fig 5 after the removal of leverage points, which were the cooking temperatures equal to or less than 100°C. The lines indicate the linear fit, and the 95% confidence intervals are presented as gray areas around each fitted line.
(TIFF)

S1 Table Other sources of purchasing oysters. (DOCX)

S2 Table Factors assessing whether oysters are ready to serve. (DOCX)

S3 Table Inputs about preparing oysters provided by the participants. (DOCX)

S1 File Biorender publication license. (PDF)

## References

[1] McLeod C, Hay B, Grant C, Greening G, Day D. Localization of norovirus and poliovirus in Pacific oysters. J Appl Microbiol. 2009;106(4):1220–30. doi: 10.1111/j.1365-2672.2008.04091.x 19187161

[2] Nappier SP, Graczyk TK, Schwab KJ. Bioaccumulation, retention, and depuration of enteric viruses by *Crassostrea virginica* and *Crassostrea ariakensis* oysters. Appl Environ Microbiol. 2008;74(22):6825–31. doi: 10.1128/AEM.01000-08 18820067 PMC2583511

[3] Wang D, Wu Q, Kou X, Yao L, Zhang J. Distribution of norovirus in oyster tissues. J Appl Microbiol. 2008;105(6):1966–72. doi: 10.1111/j.1365-2672.2008.03970.x 19120643

[4] FDA. Selecting and serving fresh and frozen seafood safely. 2024 [Accessed 2025 January 25]. https://www.fda.gov/food/buy-store-serve-safe-food/selecting-and-serving-fresh-and-frozen-seafood-safely

[5] U.S. CDC. Preventing vibrio infection. 2024 [Accessed 2025 January 25]. https://www.cdc.gov/vibrio/prevention/vibrio-and-oysters.html

[6] Parveen S, Tamplin ML. Vibrio vulnificus,Vibrio parahaemolyticusandVibrio cholerae. Guide to Foodborne Pathogens. John Wiley & Sons, Ltd. 2013: 148–76. doi: 10.1002/9781118684856.ch9

[7] Pouillot R, Smith M, Van Doren JM, Catford A, Holtzman J, Calci KR, et al. Risk assessment of norovirus illness from consumption of raw Oysters in the United States and in Canada. Risk Anal. 2022;42(2):344–69. doi: 10.1111/risa.13755 34121216 PMC9291475

[8] CDC US. BEAM (Bacteria, Enterics, Ameba, and Mycotics) Dashboard. 2025 [Accessed 2025 January 25]. https://www.cdc.gov/ncezid/dfwed/BEAM-dashboard.html

[9] British Columbia CDC. Fish & shellfish safety. 2024 [Accessed 2025 January 25]. http://www.bccdc.ca/health-info/prevention-public-health/fish-shellfish-safety

[10] Richards S, Vassalos M, Motallebi M. Factors affecting consumer purchasing decisions and willingness to pay for oysters in South Carolina. J Food Distribution Res. 2022;53:1–25. doi: 10.22004/ag.econ.339684

[11] U.S. Department of Labor. Standard Industrial Classification (SIC) system search. 2025 [Accessed 2025 January 31]. https://www.osha.gov/data/sic-search

[12] R Core Team. R: A language and environment for statistical computing. Vienna, Austria: R Foundation for Statistical Computing; 2024.

[13] Wickham H, Bryan J. readxl: read Excel files. 2023. https://CRAN.R-project.org/package=readxl

[14] Wickham H. ggplot2: elegant graphics for data analysis. 2016 [Accessed 2024 November 14]. https://ggplot2.tidyverse.org

[15] Wickham H, François R, Henry L, Müller K, Vaughan D. dplyr: a grammar of data manipulation. R package version 1.1.4. 2023. doi: 10.1007/978-1-4842-6876-6_1

[16] Neuwirth E. RColorBrewer: ColorBrewer palettes. 2022. https://CRAN.R-project.org/package=RColorBrewer

[17] Moon KW. Webr: Data and Functions for Web-Based Analysis. 2020. https://CRAN.R-project.org/package=webr

[18] Census Bureau US. U.S. Census Bureau Gazetteer Files. 2025 [Accessed 2024 November 12]. https://statics.teams.cdn.office.net/evergreen-assets/safelinks/1/atp-safelinks.html

[19] U.S. Census Bureau. Cartographic Boundary Files - Shapefile. Census.gov. 2024 [Accessed 2025 May 13]. https://www.census.gov/geographies/mapping-files/time-series/geo/carto-boundary-file.html

[20] USDA National Agricultural Statistics Service (NASS). 2023 Census of Aquaculture. 2024. https://www.nass.usda.gov/Publications/AgCensus/2022/Online_Resources/Aquaculture/index.php

[21] Hanson TR, House L, Sureshwaran S, Posadas BC, Liu A. Opinions of US consumers toward oysters: results of a 2000-2001 survey. 2016. https://repository.library.noaa.gov/view/noaa/13654

[22] Mirmahdi RS, Dicker S, Farzad R, Andrew M, Simonne A. Ensuring safe oysters: Essential handling, preparing, and cooking practices: FSHN25-8/FS470. 2025. 10.32473/edis-fs470-2025

[23] Chen H, Wang M, Yang C, Wan X, Ding HH, Shi Y, et al. Bacterial spoilage profiles in the gills of Pacific oysters (*Crassostrea gigas*) and Eastern oysters (*C. virginica*) during refrigerated storage. Food Microbiol. 2019;82:209–17. doi: 10.1016/j.fm.2019.02.008 31027776

[24] Degner RL, Petrone C. Consumer and restaurant manager reaction to depurated oysters and clams. 94–1. Florida Agricultural Market Research Center, IFAS, University of Florida; 1994. https://ufdc.ufl.edu/uf00026922/00001

[25] Li T, Kecinski M, Messer KD. Heterogeneous preferences for oysters: evidence from field experiments. Agric Resour Econom Rev. 2017;46(2):296–314. doi: 10.1017/age.2017.16

[26] Tulshian N, Wheaton F. Oyster (*Crassostrea Virginica*) shell thermal conductivity: technique and determination. Transactions of the ASAE. 1986;29(2):0622–5. doi: 10.13031/2013.30203

[27] Wheaton F. Review of oyster shell properties. Aquacultural Engineering. 2007;37(1):14–23. doi: 10.1016/j.aquaeng.2006.11.002

[28] Galtsoff PS. The *American oysters*, Crassostrea virginica gmelin. Fishery Bull. 1964.

[29] Leung J, BCIT School of Health Sciences, Environmental Health, Andraza C, McIntyre L, Heacock H. Evaluation of internal temperature of oysters following standard thermal process recipes. EPHJ. 2018. doi: 10.47339/ephj.2018.64

[30] Mirmahdi RS, Montazeri N. Progress and challenges in thermal inactivation of norovirus in oysters. Crit Rev Food Sci Nutr. 2025;:1–14. doi: 10.1080/10408398.2025.2467209 40007190

