# Supplementary figures and images for: Oyster cooking practices in the United States-based restaurants—A survey
Source: PLoS One. 2025 Jul 16;20(7):e0327330. doi: 10.1371/journal.pone.0327330 (PMC12266452; doi:10.1371/journal.pone.0327330)

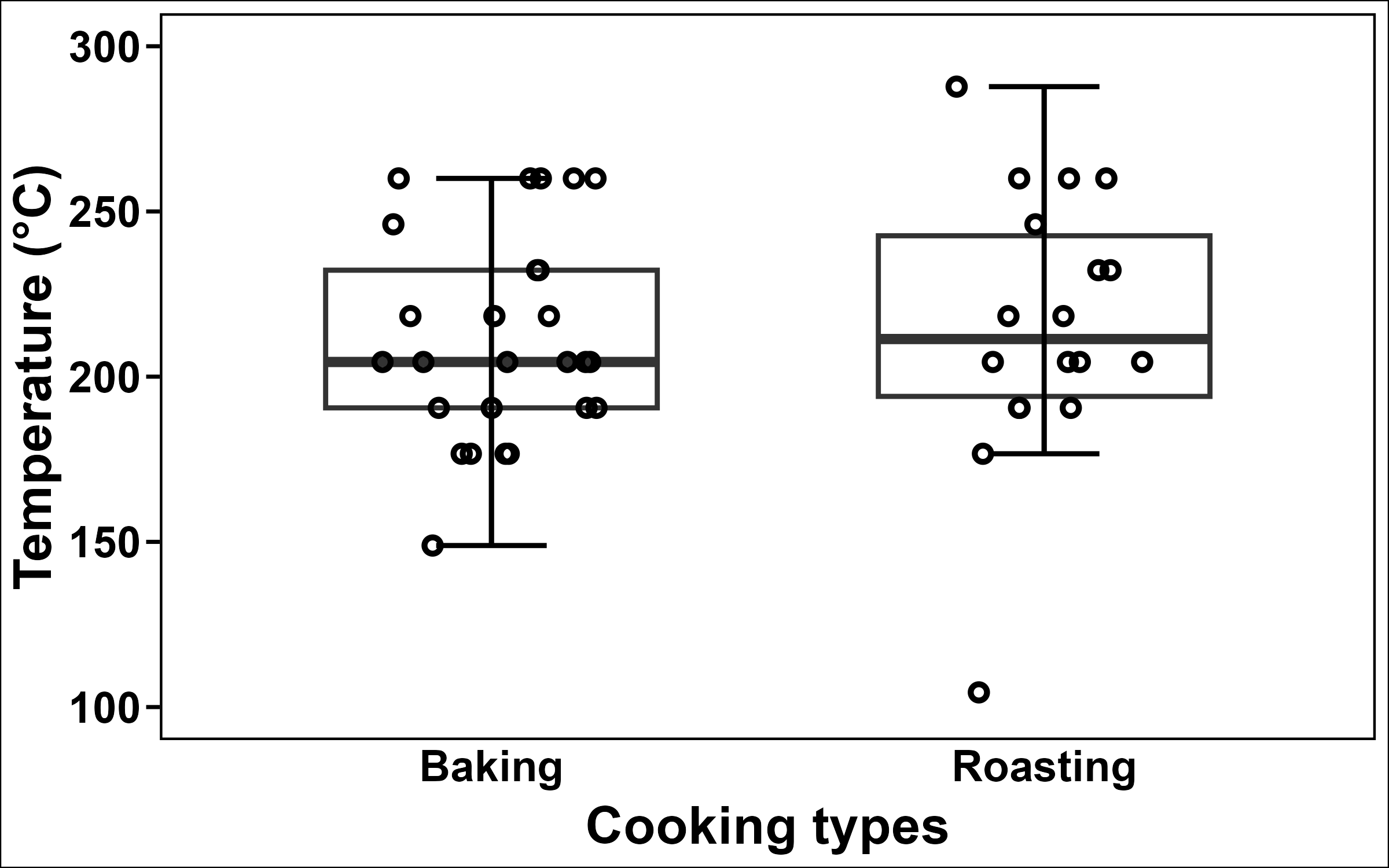

Supplement: S1 Fig — Box plots illustrate the cooking temperatures restaurants reported to use for various oyster cooking techniques. The temperature for steaming was assumed to be 100ºC (not shown). The upper line in each box represents the third quartile, the middle line indicates the median, and the lower line corresponds to the first quartile. (TIFF) [file pone.0327330.s001.tiff]

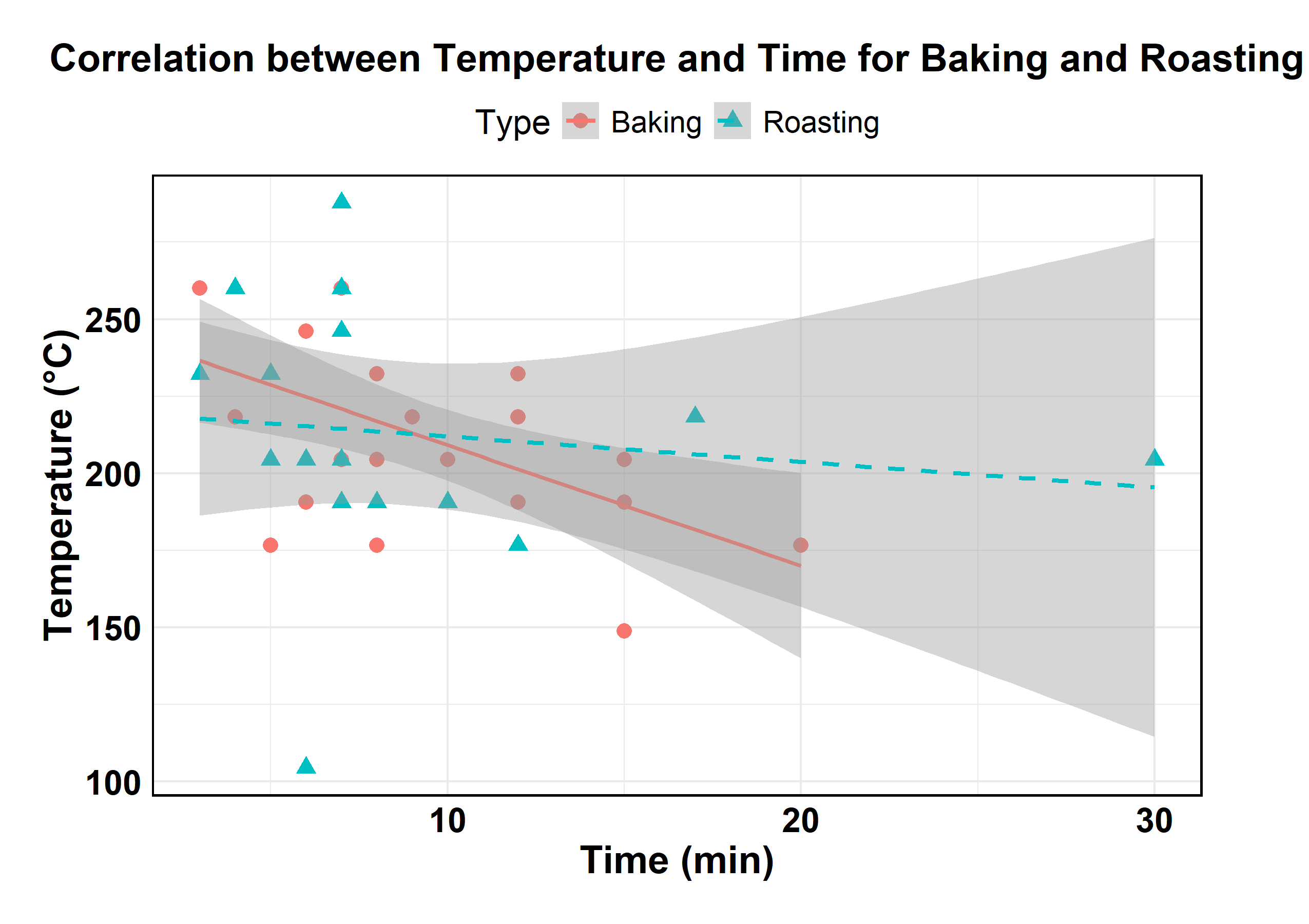

Supplement: S2 Fig — Data represents the recalculation of Fig 5 after the removal of leverage points, which were the cooking temperatures equal to or less than 100°C. The lines indicate the linear fit, and the 95% confidence intervals are presented as gray areas around each fitted line. (TIFF) [file pone.0327330.s002.tiff]
