# Supplementary material for: Oyster cooking practices in the United States-based restaurants—A survey
Source: PLoS One. 2025 Jul 16;20(7):e0327330. doi: 10.1371/journal.pone.0327330 (PMC12266452; doi:10.1371/journal.pone.0327330)
Supplement: S1 Table — (DOCX) [file pone.0327330.s003.docx]

**S1 Table. Other sources of purchasing oysters**

| A.4.5 |
| --- |
| Anderson seafood Distributor |
| food service |
| A company named Kellum |
| wholesale distributor |
| Oyster processing company that buys from harvester. |
| Cisco, major food distributors like US foods or Cisco |
| a Processor |
| seafood distributor |
| WHOLESALE FISH PROVIDER |
| Seafood supply company |
| fish seller and buyer |
| farms and wholesalers |
| major big food company |
| US based and Sam brunch |
| Distributer |
| Sisco or us foods |
| seafood trucker - involved in all the farming |
| oyster coop |
| Wholesaler in Houston |
| food distributor |
| cisco |
| wholesale distributor |
| food service company |
| seafood supplier that purchases from a harvester |
| Food distributor (Cisco, Louisiana Seafood) |
| food performance group |
| Distributor |
| Food purveyor |
| vendor |
| Seafood purveyor |
| Distributer / wholesaler |

*Refer to the questionnaire provided in Table. 1 of the manuscript.
