# Supplementary material for: Oyster cooking practices in the United States-based restaurants—A survey
Source: PLoS One. 2025 Jul 16;20(7):e0327330. doi: 10.1371/journal.pone.0327330 (PMC12266452; doi:10.1371/journal.pone.0327330)
Supplement: S2 Table — (DOCX) [file pone.0327330.s004.docx]

**S2 Table. Factors assessing whether oysters are ready to serve**

| B.5 | B.9 | B.13 |
| --- | --- | --- |
| Shell open, and texture | Hot, the texture is well. timer used. the smell too | None of those, they bubble on grill, coating gets brown |
| Texture, visually see if it is ready. they’re hot | Texture and juiciness | Texture and the juiciness. the toppings are fully melted |
| all of those above | Color | Color |
| Texture and color | All | Color |
| Appearance | Similar to steamed, Will shrivel up, have a different look, can be golden brown. Depends on how the customer wants it. Color varies by customer order. | We don't do that |
| all the above | Texture | Definitely shell open, texture, color, and temperature |
| It will shrivel, it will look wrinkly, become light golden brown | Texture | Liquid boil |
| Shell openness | Texture | Opacity, juiciness |
| Open | When edges curl up | There’s a color they turn that are more solid in color and firm in texture |
| Openness | Juiciness | Texture and color |
| Texture | Color | All of the above |
| All of the above. We do the oysters in a steam box. For Oyster Rockefeller | Color, dryness, thermo | appearance |
| Texture and color | Cheese is melted; the oysters are well done | We don’t let them dry out too much. juice in shell. cook it to reduce it to half |
| size the meat, shrinks | Texture and color | Internal temperature |
| It will be temperature is right. Make sure not overdone not too rubbery. The appearance as well. It shouldn't be shriveled up. | texture and color | Color |
| Texture and color | Color and texture | Toasted topping, edges, boiling temp liquor |
| shell open, smell | Thermo | Texture |
| guest preference | Liquid bubbling | Texture |
| Firmness | Juice boil | Juiciness |
| A health code timer. We have to cool them for 3 and a half minutes, so we put a timer on. | Thermometer | Texture and size change |
| Internal temperature | Toppings brown, temp | Use of instant read thermometer to determine safe cooked temperature |
| all of the above | All of the above | Color |
| shell openness opacity | Shell open, sit under heat, once is free of moisture, gills start to show, and oyster pumps up | When the shell starts to open. |
| Open | all of the above |  |
| Texture | Texture |  |
| Time | Internal temperature |  |
| Shell openness and juiciness | all |  |
| Texture, color, use of instant thermometer | Crispy toasted, boiling the garlic butter, important boiling |  |
| Texture | open |  |
| Once they start curling up on the side. | Texture |  |
|  | Texture |  |

*Refer to the questionnaire provided in Table. 1 of the manuscript.
