# Supplementary material for: Oyster cooking practices in the United States-based restaurants—A survey
Source: PLoS One. 2025 Jul 16;20(7):e0327330. doi: 10.1371/journal.pone.0327330 (PMC12266452; doi:10.1371/journal.pone.0327330)
Supplement: S3 Table — (DOCX) [file pone.0327330.s005.docx]

**S3 Table.** Inputs about preparing oysters provided by the participants

| B.15* |
| --- |
| We keep logs to make sure everything is in date |
| You have to clean them a bit to get parasites, shells, and pearls out of them. |
| Store before they are cooked, on ice |
| When we bake them, we bake with cheese and garlic butter. we rent them before we serve them |
| We add spices after boil. |
| We also fry oysters to prepare them. |
| Use spices. Cooking, we use regular utensils. opening we determine if the oyster is good and bad. Sometimes they are bad just by looking at them before they are cooked. if an oyster is open, it’s a bad oyster |
| Baked oysters are baked with garlic, butter, cheese, and bacon. |
| Pan fried oysters are probably the biggest seller. we do pan fried oyster as an appetizer and a pan-fried oyster fettucine entree |
| The cookware in particular is called Rationales. We use rationales and anybody doing the survey should understand what that is |
| We fry oysters as well |
| Use corn flour, evaporated milk, seasonings |
| holding temperature and where they were held |
| Keep them as cold as you can |
| Just bread them and fry them |
| maintain proper temperature |
| When preparing oysters, first we clean the shells, we water down the shells, then we ice them down, then we scrape the top shell off and the oyster from the bottom shell. We use salt water and spray the oyster to get rid of any extra shells or grit so that customers get clean, fresh, and salty oysters. We keep the oyster muscles on the bottom shell. |
| Honey, Cheese fruit |
| We have a homemade batter that we deep fry oysters in |
| roll flour deep fry |
| chargrilled blend of spices they use |
| batter them, flour, own batter mix, |
| most is raw |
| raw as well |
| make sure the oyster has been salt washed before cooking. Salt dip is very important |
| Working with raw oysters, people need to make sure they have gloves on. We track all of our batches in case we get a bad batch, we can determine which batch it came from. |
| When frying the oysters we typically use batter using flour and seasoning which protects it from over seasoning. People do not like dry or chewy oysters, so we try not to overcook them. Oysters can be undercooked or served raw and are still considered safe for consumption |
| Steam them and serve them, it's pretty standard |
| Thermometer |
| Bread is straightforward, do not need a thermometer all pre shucked |
| Smell and open partial and check temp |
| butter and cheese |
| not just fry |
| batter and fry |
| shuck to order, put on ice, pre shuck them |
| tags, freshness and quality, shucked within proper time |
| Cheese, butter and different toppings, sauces |
| All our oysters are freshly cleaned before we prepare them. |
| We scrub them first. And we also fry oysters. |
| We always have our temperature controlled. And they always check every order. |
| Charr grilled cooked on open flame, also fry pre shucked, fry them for 2 minutes |
| Top them with butter cheese garlic parsley |
| Put butter combo, cheese, spinach |
| we serve a lot of raw oysters |
| We use different spices because we are a Cajun restaurant, so they are fried in platters with a cornmeal-based batter, and they are fried whole. we do chargrill as well on the half shell and that is usually with a butter sauce |
| butter garlic and parmesan before putting on grill and they shuck their oyster |
| We also fry them |
| fry breading, dip flour then washes, before fried |
| Evaporated milk, batter, sifted and grease change daily only fryer for just for oyster |
| cleaning the oyster |
| Broiling the heat comes to the top, burns oyster when using heat from oven better to have convection oven |
| Sanitation is very, very important in cooking oysters. |
| Yes, raw |
| The cleaning of them is one of the most important parts. You have to be careful with the shells and everything. |
| Butter garlic cheese bacon onion peppers |
| We typically cook them only for oyster Rockefeller or fried oysters. |
| We use a variety of ingredients as well as the gulf oysters |
| Not sure why you don't ask about fried or even raw oysters. Raw oysters are probably the most popular way to serve oysters. On the previous question I selected "other" not because we don't use a thermometer. It is because we don't serve our oysters any of the three ways you have listed. |
| butter, garlic, parmesan cheese |

*Refer to the questionnaire provided in Table. 1 of the manuscript.
